# Book Review: What Matters? Putting Common Sense to Work

**DOI:** 10.3389/fpsyg.2017.00264

**Published:** 2017-02-24

**Authors:** Gavan Lintern

**Affiliations:** Monash University Accident Research Centre, Monash UniversityMelbourne, VIC, Australia

**Keywords:** cognitive theory, pragmatism, cognitive design, abductive logic, rationality

In What Matters? John Flach and Fred Voorhorst offer an approach to understanding every-day cognition that stands in contrast to the standard approach that has generated interest in logical puzzles, bias, and formal approaches to rationality. More on that question mark in the title later, but the words presage a concern with what matters to us as cognitive agents as we engage purposefully with our world and what we, as cognitive scientists and designers of cognitive support systems, should attend to as we build a science of purposeful cognitive engagement with the world. In building their case, Flach and Voorhorst interlace four main themes.

The first is a metaphysical argument that cognition is not a mental activity but rather, is a functional interaction between us and our environment as we cope with the challenges of normal life. Flach and Voorhorst conceptualize cognition as a triadic system of a structured world, of information that specifies that structure, and the functional significance of that structure for us as we engage purposefully with our world. Cognitive experience encompasses a coupling between specific structural properties of our environment and our own capabilities as mediated by information that specifies that structure. Where we can interact productively with those structural properties, they become functional properties, otherwise viewed as affordances, and thereby become meaningful. What matters (what we as cognitive agents need to know to lead a satisfying and productive life) can only be understood through an analysis that spans mind and matter and that attends to the coupling between them. The focus moves from formal descriptions of the world, those that rely on standard scientific dimensions, to descriptions in terms of what the world affords us.

The second theme focuses on the role of experience in shaping cognition. This theme is aligned with Pragmatics, that branch of Philosophy that studies the ways in which experience in the world contributes to meaning. Thus, the meaning of cognitive content (its semantics) is pragmatically grounded by virtue of being discovered through interactive engagement with the world. Cognition is not a product of brain activity but emerges from functional interactions involved in coping with the complexities of day-to-day life. This coping is adaptive, where the consequences of action are fed back as information, and is circular, where actions both shape and are shaped by consequences.

The third theme focuses on the nature of rationality. Flach and Voorhorst distinguish the formal logics of deduction and induction from the pragmatic logic of abduction; a form of logical inference from observation to explanation. In contrast to deduction and induction, which describe syntactical relations, abduction describes a semantic relationship essential to creativity and insight. Abductive logic involves learning through assimilation of past experiences, which leads to generation of beliefs about our interaction with the world. Those beliefs are evaluated through action, and revised through accommodative processes associated with the feedback from that action.

Historically, cognitive science has taken rationality, defined as conformance to formal logic, as a standard of the ideal. Deviations are viewed as cognitive error or cognitive bias. Flach and Voorhorst observe that, at least for human cognition, rationality might better be viewed pragmatically. Does the behavior in question work in practice? Does it lead to satisfying consequences? Pragmatically, a rational system is one that learns from its mistakes and adapts toward increasingly stable relations with its ecology. An irrational system is one that fails to adapt either by responding inappropriately to the feedback or by ignoring it to make the same mistakes over and over again.

Notably, and in contrast to deductive logic, abductive logic does not lead to a syntactically verifiable conclusion. Rather, it leads to a best guess or a best inference. In the abductive logic scheme, life is not about making the right choices but about heading in a direction and selecting from the opportunities we generate or encounter on the way. For Flach and Voorhorst, life is an extended process of *muddling through*.

The final theme focuses on implications for design. With their emphasis on formal computational models and closed-form domains, theories in cognitive science sometimes seem to be little more than a plausible cover story. Can we develop an approach to cognition and learning with design imperatives that can actually improve performance in complex, unpredictable and untidy work domains? Given the view that life is an extended process of muddling through, *the ultimate goal of design should be to engage humans more effectively in order to facilitate better muddling* [p. 345]. Flach and Voorhorst offer us a coherent and comprehensive treatment of a cognition and learning that can help us understand how we act and interact in our world and how we might design technologies to support our purposeful and meaningful engagement with that world.

To conclude, what matters, the meaning in a domain that can be employed within the context of purposeful action, is an emergent property of the closed-loop coupling between mind (awareness) and matter (ecology). And now, back to that question mark in the title. The book is not so much an account of what matters but an inquiry. It advances an ongoing exploration into what matters within the context of human experience; an exploration we are unlikely to complete any time soon. Science itself is a process of muddling through. Flach and Voorhorst have not so much told us what matters but rather, have outlined the conceptual tools that will allow us to engage in our own exploration of what matters for ourselves and for our scientific endeavors.

## Author contributions

The author confirms being the sole contributor of this work and has approved it for publication.

### Conflict of interest statement

The authors declare that the research was conducted in the absence of any commercial or financial relationships that could be construed as a potential conflict of interest.

